# Studies on Pitting Corrosion of Al–Cu–Li Alloys Part III: Passivation Kinetics of AA2098–T851 Based on the Point Defect Model

**DOI:** 10.3390/ma12121912

**Published:** 2019-06-13

**Authors:** Elmira Ghanbari, Alireza Saatchi, Xiaowei Lei, Digby D. Macdonald

**Affiliations:** Department of Materials Science and Engineering, University of California at Berkeley, Berkeley, CA 94720, USA; elmira.ghanbari@berkeley.edu (E.G.); alireza.saatchi@berkeley.edu (A.S.); leixw1987@gmail.com (X.L.)

**Keywords:** Al–Cu–Li alloys, point defect model, mixed potential model, passivation kinetics

## Abstract

In this paper, the passivation kinetics of AA2098–T851 was investigated by a fundamental theoretical interpretation of experimental results based on the mixed potential model (MPM). The steady state passive layer formed on the AA2098–T851 in NaHCO_3_ solution in a CO_2_ atmosphere upon potentiostatic stepping in the anodic direction followed by stepping in the opposite direction was explored. Potentials were selected in a way that both anodic passive dissolution of the metal and hydrogen evolution reaction (HER) occur, thereby requiring the MPM for interpretation. Optimization of the MPM on the experimental electrochemical impedance spectroscopy (EIS) data measured after each potentiostatic step revealed the important role of the migration of Al interstitials in determining the kinetics of passive layer formation and dissolution. More importantly, it is shown that the inequalities of the kinetics of formation and dissolution of the passive layer as observed in opposite potential stepping directions lead to the irreversibility of the passivation process. Finally, by considering the Butler–Volmer (B–V) equation for the cathodic reaction (HER) in the MPM, and assuming the quantum mechanical tunneling of the charge carriers across the barrier layer of the passive film, it was shown that the HER was primarily controlled by the slow electrochemical discharge of protons at the barrier layer/solution (outer layer) interface.

## 1. Introduction

The third-generation Al–Li alloy AA2098 was initially introduced by Alcoa in 2011. The beneficial effects of alloying aluminum with Li are increased stiffness and decreased density that has made this alloy an appropriate candidate for the use in aerospace applications [1,2,3,4]. While there have been numerous studies focused on the corrosion properties of Al and its alloys, the electrochemical properties of the relatively new Al–Li AA2098 have not been investigated to a significant extent. It is well known that Al alloys are prone to pitting corrosion, which mostly depends on the chemistry of the environment [5,6,7,8,9,10,11]. The breakdown of the protective barrier layer on the metal surface in the presence of aggressive anions, such as Cl^−^, leads to the initiation of the pitting corrosion [12]. In previous papers of this series (paper I and II), passivity breakdown and pit initiation on AA2098–T851 along with two other Al alloys with comparable composition, except for Li content, were investigated using the potentiodynamic polarization technique and damage morphology characterizations. AA2098–T851, which had higher Li content than the other two alloys (AA2029–T8 and AA2060–T8), showed the best pitting resistance behavior [13,14]. Using the point defect model (PDM), the near-normal distribution of breakdown potential of AA2098–T851 for one [Cl^−^] was optimized and the optimized model parameters were used to predict the distributions for other [Cl^−^] concentrations. The predicted distributions were found to be in close agreement with experimental results [14]. However, no further insight in regard to the passivation kinetics and underlying mechanisms of the phenomena was provided by the previous papers. Different mechanisms have been proposed in the literature for explaining the passive layer formation and its breakdown on metallic structures [15,16,17,18,19,20]. Of the most prominent models in the field of passivity, the high field model (HFM), which considers the cation interstitial migration in the passive layer, and the place exchange model (PEM), which assumes both cation/anion exchange as the mechanism of film growth have been extensively used [15,18]. Neither of these models explains the observed bilayer structure of the passive film or the existence of a steady-state in the barrier layer thickness and the passive current density, and hence are at odds with experimental results. However, the point defect model (PDM) accounts for both bilayer passive film structure and the steady-state current and film thickness, as well as for the transients in these properties in response to changes in various independent variables [19,20]. This model predicts that the steady-state barrier layer thickness should display a linear dependence on the applied potential and pH and these relationships have been confirmed by numerous empirical observations. According to the PDM, the passive film formation is controlled by the kinetics of the point defects generation and annihilation at the metal/barrier layer (m/bl) and the barrier layer/outer layer (bl/ol) interfaces. A newly modified PDM, the mixed potential model (MPM), considers the influence of the partial cathodic reaction on the passive layer properties in addition to the partial anodic process [21].

It has been reported that the kinetics of passivation (i.e., the rate with which the passive state is formed) depend on the potential stepping direction [21]. This inequality of certain kinetic processes leads to the irreversibility of the metallic passivation in opposite potential scanning directions. To be able to predict the corrosion damage of the Al–Li 2098, it is necessary to understand the passivation kinetics and its irreversibility. As will be explained in this paper, by applying the MPM, we have shed light on understanding the irreversibility kinetics of the passive film formation on Al–Li 2098 in the sodium bicarbonate buffer solution.

## 2. Materials and Methods

### 2.1. Electrode and Solution

In this paper, AA2098–T851 coupons were mounted in epoxy resin with an exposed surface area of 0.35 cm^2^. The nominal chemical composition of the AA2098–T851 alloy is: Cu 3.71 wt%, Li 1.29 wt%, Mg 0.26 wt%, Mn 0.03 wt%, Ag 0.03 wt%, Zr 0.06 wt%, Zn 0.01 wt%, with the balance being Al.

A copper wire glued with a conductive adhesive was used for the electrical connection. The preparation of samples included grinding with SiC sandpapers with grit number from 400 to 1200 followed by rinsing with acetone, ethanol, and double distilled water and drying with N_2_ gas.

Experiments were performed in triplicate in analytical grade reagents 0.1 M NaHCO_3_ buffer solution with the pH of 6.7 at room temperature (25 °C) under atmospheric pressure of CO_2_, to simulate the surrounding environment of space vehicles since aircraft fly through the atmosphere that contains CO_2_. Moreover, it is an effective buffer system that does not introduce species that are not in contact with Al–Li alloys in the field. For complete deaeration of the solution with pure CO_2_ gas, the solution was sparged at least 1 h before an experiment, and sparging was continued throughout an experiment.

In a three-electrode cell, AA2098–T851 alloy was used as the WE, an SCE was employed as the RE, and a platinum mesh served as the CE, for performing electrochemical experiments. Additionally, for preventing contamination of the working solution by leakage from the RE, the SCE was contained in a separate compartment that maintained electrolytic contact with the cell via a Luggin capillary.

### 2.2. Electrochemical Set-up

A Gamry Electrochemical Measurement System (PC3, Warminster, PA, USA) potentiostat was used for electrochemical experiments in this work. Before each experiment, a potential of −1 V_SCE_ was applied for 10 min in order to establish a reproducible initial state.

In order to define the passive range of AA2098–T851 in 0.1M NaHCO_3_/CO_2_(g) buffer solution, potentiodynamic polarization (PDP) at a scan rate of 0.166 mV/s scan rate was employed. Based on the passive region obtained from the PDP curve, film formation potentials of −600, −300, 0, 300 and 600 mV_SCE_ were selected for conducting potentiostatic polarization. The anodic potentiostatic step potentials, starting from −600 mV_SCE_, were followed by cathodic potential stepping from 600 mV_SCE_ ending at −600mV_SCE_. Each potentiostatic polarization lasted for 24 h in order to obtain a steady-state passive film. After each step, two successive electrochemical impedance spectroscopy (EIS) analyses were performed using a 10 mV sinusoidal excitation around the applied potential (*E_app_*), with the frequency being scanned from 100 kHz down to 0.01 (high to low frequency) and from 0.01 to 100 kHz (low to high frequency).

## 3. Results

### 3.1. Potentistatic Polarization

**Figure 1:**
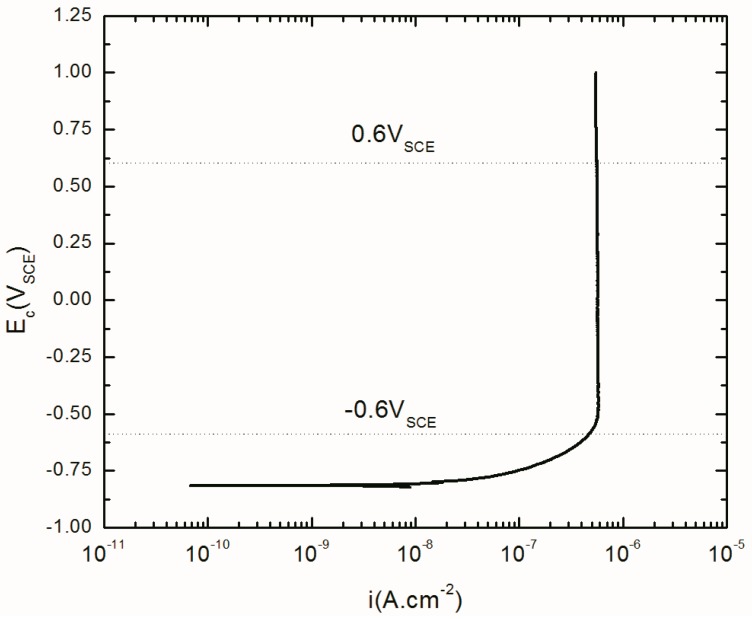
Polarization curve of AA2098–T851 alloy in 0.1M NaHCO_3_ /1 atm of CO_2_ at 25 °C.

Figure 1 shows the potentiodynamic polarization curve of AA2098–T851 in 0.1 M NaHCO_3_ in contact with CO_2_ at atmospheric pressure. As can be seen in this figure, there is a broad range of passivity starting from −0.6 V_SCE_ to more than 1.00 V_SCE_. Therefore, the passive range from -0.6 V_SCE_ to 0.6 V_SCE_ (marked by the dashed lines in the figure) was selected for the study of the passivation kinetics in this paper.

**Figure 2:**
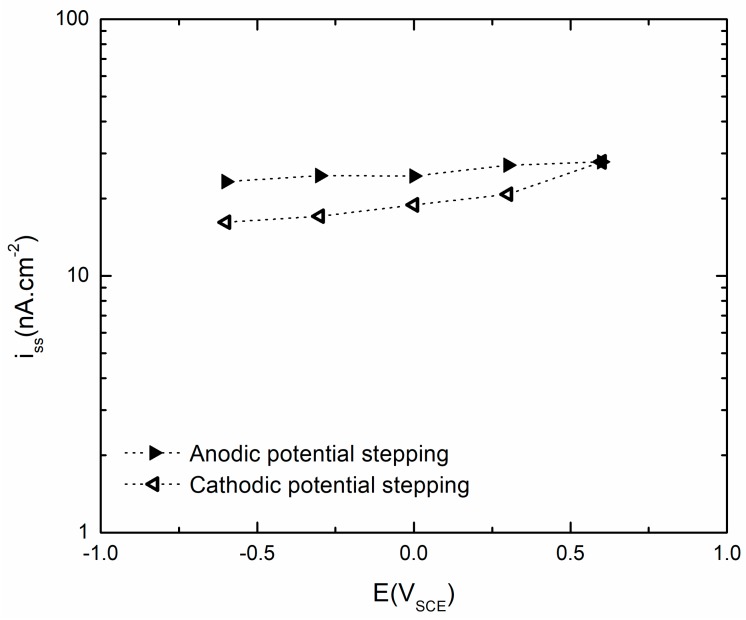
Steady-state current density for both anodic and cathodic potential stepping directions.

Figure 2 shows a typical steady-state potentiostatic current densities obtained at different potentials after 24 h of polarization of AA2098–T851 alloy in 0.1 M NaHCO_3_ buffer solution in contact with CO_2_ gas at atmospheric pressure. According to the PDM, the value of the logarithm of the steady-state current density (*i_ss_*) for an n-type semi-conductor, in which no change occurs in the oxidation state of the cation upon ejection from the barrier oxide layer, is independent of *E_app_* (as shown in Equation (1)), but that the relationship should be linear if the barrier layer is p-type [22]. Equation (1) shows variation of the *i_ss_* with *E_app_* for an n-type barrier layer with δ≠χ [22].

(1) $${\left(\frac{\partial \log i_{ss}}{\partial E}\right)}_{pH,C_{Al}^{3+}}=\frac{\alpha \alpha_{7}\left(\delta -\chi \right)\gamma }{2.303}$$

where *α* is the polarizability constant that correlates the potential drop across the barrier layer/ solution (bl/s) interface to the applied potential (*E_app_*), *χ* is the stoichiometry of the oxide (MO_χ/2_), δ is the oxidation state of the metal in the (ol/s) interface and $\gamma =\frac{F}{RT}$. For the passive film on Al, δ=χ so that ${\left(\frac{\partial \log i_{ss}}{\partial E}\right)}_{pH,C_{Al}^{3+}}$ = 0, as is observed approximately in Figure 2. As reported by others, the barrier layer of the passive film on Al and its alloys is unequivocally an n-type semiconductor [17,23,24,25,26,27]. Even so, Figure 2 shows that the steady-state current density slightly increases with an increasing anodic potential (15–28 nA cm^−2^), and there is a dependence upon the direction of the change in potential. We postulate that this slight dependence is a consequence of residual irreversibility in the formation/dissolution of the barrier layer (bl) as the potential is stepped in the increasing and decreasing directions, as will be discussed below.

It is important to note that the observed current density comprises both anodic and cathodic components. Although the partial anodic current density is predicted to be independent of *E_app_*, there exists a competition between partial anodic and partial cathodic currents in determining the total (observed) current [21]. This observation has been made by other authors [21,28] and shows the importance of considering the cathodic current produced by hydrogen evolution due to the water reduction in deaerated condition when considering the electrochemistry of reactive metals, such as Al, Li, Mg, and Zn.

Also, Figure 2 shows that the value of the passive current density during potentiostatic polarization in the cathodic potential step direction is lower than that observed in the opposite (anodic stepping) direction. This could be due to the persistence of the previously formed passive layer at higher potentials that led into the higher charge transfer resistance during cathodic potential stepping direction (i.e., due to irreversibility in the anodic partial process of film formation). This residual barrier layer could hinder transport of species across the film and result in a lower current density compared to that in the anodic direction. Alternatively, this layer might reflect a lower tunneling probability of electronic charge carriers across the barrier layer to the cathodic reaction center located at the bl/s interface, due to a slightly thicker bl on the reverse potential stepping direction compared with that for the forward stepping direction. It is also expected to have a significant effect on the kinetics of the cathodic partial process, because the quantum mechanical tunneling probability of charge carriers from the metal to the cathodic reaction center at the bl/s interface is a sensitive function of the barrier layer properties, especially of the thickness [29,30].

### 3.2. Impedance Analysis of the Passive Layer

**Figure 3:**
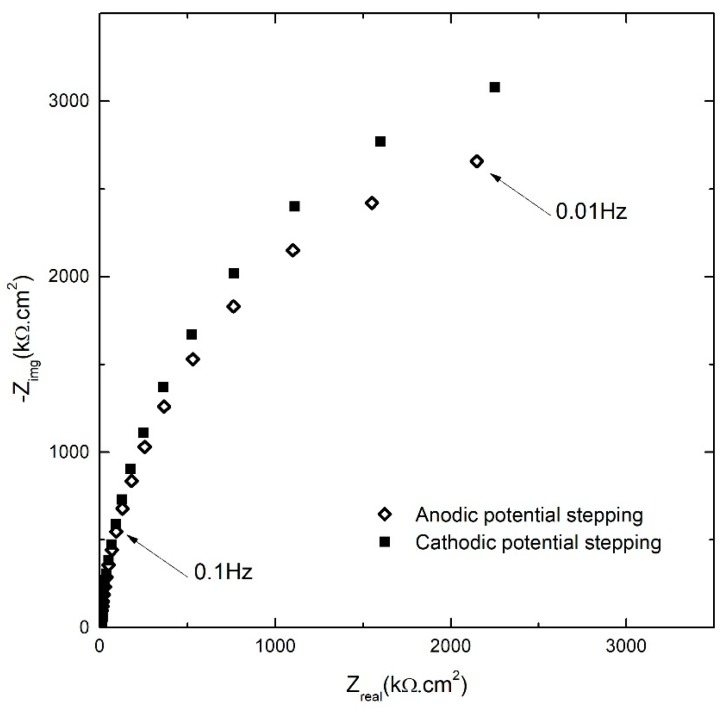
Nyquist plots of the AA2098–T851 alloy in 0.1 M NaHCO_3_ /1 atm of CO_2_ at 25 °C for both anodic and cathodic potential stepping directions at +300 mV_SCE_ [31].

EIS analysis was performed after each potentiostatic potential step in order to analyze the kinetics of the reactions that occur during barrier layer formation and dissolution. Figure 3 shows the Nyquist plots at +300 mV_SCE_ that were obtained in both the anodic and cathodic potential stepping directions.

This figure shows typical behavior of the passive layer that has an arc-shape with small curvature and significant magnitude [32]. Another feature in this figure to be noted is that at all frequencies the values of both the real (*Z_real_*) and imaginary (*Z_img_*) components of the impedance in the cathodic stepping direction were higher than that in anodic stepping direction. While there is a little difference in the *Z_real_* in two opposite stepping directions, the difference of *Z_img_* values in cathodic potential stepping followed immediately after the opposite direction potentiostatic experiments, showed an increasing trend with decreasing the frequency. In other words, polarization in the cathodic potential stepping direction has a higher imaginary component of the impedance (lower capacitance) of the passive layer compared to that at the same potential in the anodic potential stepping direction. Because the geometric capacitance varies inversely with barrier layer thickness, this result is consistent with the film retaining the higher thickness established at the maximum potential on the anodic potential stepping direction upon stepping in the cathodic potential direction, as has been found to be the case for iron [28]. This was supported by the lower passive current densities in the system during cathodic potential stepping (Figure 2) and further analysis of the EIS results (which will be presented in the following sections). Thus, the observed irreversibility is attributed to the faster growth rate of the barrier layer upon anodic stepping and the lower dissolution rate of the barrier layer upon stepping in the cathodic potential direction. Parenthetically, it should be noted that the validity of all of the impedance data analyzed in this paper was confirmed by Kramers-Kronig transformation as is noted below.

**Figure 4:**
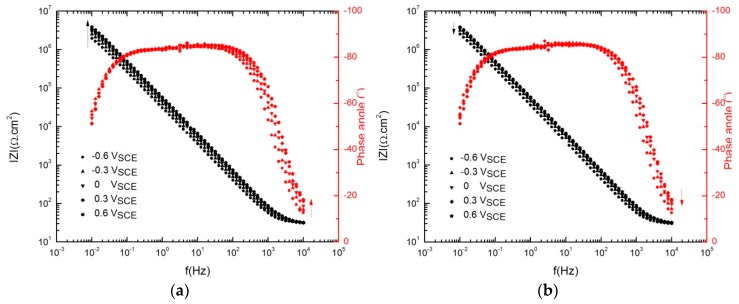
Electrochemical impedance spectroscopy (EIS) spectra of AA2098–T851 alloy after 24 h of polarization in contact with 1 atm CO_2_ (pH = 6.7, 25 °C) with high-to-low frequency scan, (**a**) anodic potential stepping direction, (**b**) cathodic potential stepping direction.

The Bode plot in Figure 4 overlays the EIS spectra for all applied potentials in both the anodic and cathodic potential stepping directions. The Bode magnitude plots in Figure 4a (black data points) demonstrate a small increase in the impedance magnitude (|*Z*|) of the film with increasing formation potential from −600 mV_SCE_ to +600 mV_SCE_ (note that the potential stepping direction is indicated by the arrows). Similarly, there was a small decrease in |*Z*| upon cathodic potential stepping from +600 mV_SCE_ to −600 mV_SCE_ (Figure 4b).

Also, the Bode phase plots in Figure 4 (red data points) show that with decreasing the frequency to 100 Hz, the phase angle increased to about −80° and remained almost constant to lower frequencies at frequencies as low as 0.1 Hz. These observations in both impedance magnitude and phase angle are a reflection of the progressive formation of the protective barrier layer with the more positive applied potential [32].

**Figure 5:**
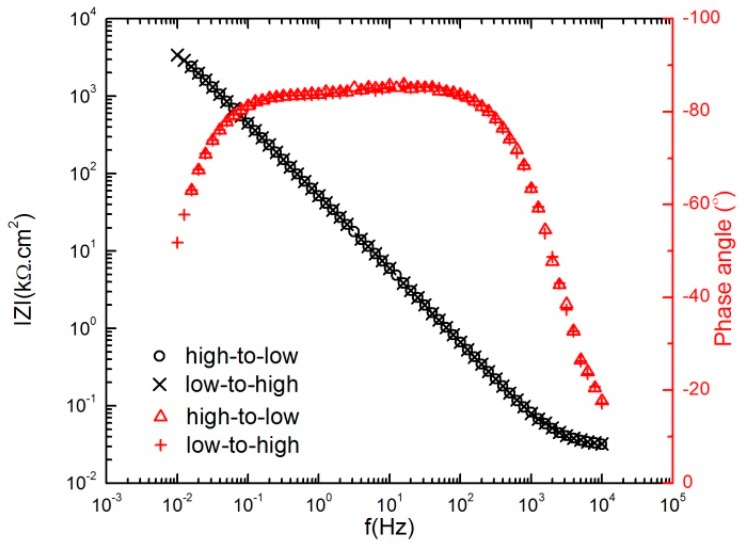
Spectra of the AA2098–T851 alloy after 24 h of polarization at 300 mV_SCE_ in contact with 1 atm CO_2_ (pH = 6.7, 25 °C), including both high-to-low and low-to-high frequency scans during anodic potential stepping.

**Figure 6:**
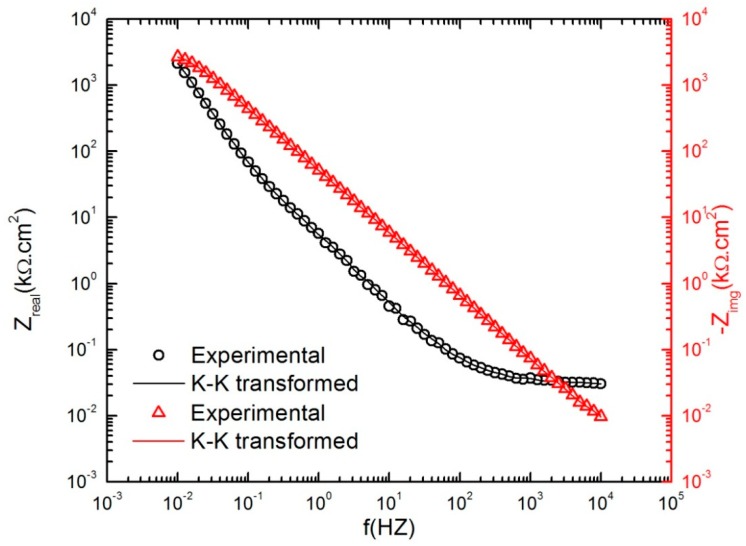
Agreement of the experimental and Kramers–Kronig transforms of the real and imaginary component of the impedance data of the AA2098–T851 alloy after 24 h of polarization at 300mV_SCE_ in contact with 1 atm CO_2_ (pH = 6.7, 25 °C) during the anodic potential stepping.

To validate the impedance data (linear system), it is necessary to demonstrate that the system complies with the constraints of linear system theory; that is, with the linearity, stability, and causality constraints, which is particularly important for an active metal like aluminum [33,34]. This was done by both experimental and theoretical analyses. It turns out that the Kramers–Kronig (K–K) transforms are not very sensitive to violation of the stability constraint [35], so that an experimental approach (Figure 5) was needed to demonstrate the stability compliance, whereas the K–K analysis (Figure 6) adequately demonstrates compliance with the linearity and causality constraints.

Figure 5 compares the EIS spectrum scanned from high-to-low frequency with that obtained immediately afterwards from low-to-high frequency after 24 h polarization at 300 mV_SCE_ during anodic potential stepping in 0.1 M NaHCO_3_ in a CO_2_ atmosphere. Since, in a steady state system, the current density and thickness of the passive layer are both time-independent [21], the good agreement of the EIS results in Figure 5 validates the stability of the system.

Additionally, the quality of the EIS data was assessed with the Kramers–Kronig (K–K) transforms. Figure 6 compares the calculated K–K transforms of the real and imaginary components of the EIS results obtained after 24 h of polarization at 300 mV_SCE_ during anodic potential stepping in 0.1 M NaHCO_3_ in contact with CO_2_ at 1 atm pressure. Agreement of the experimental EIS spectra with those calculated using the K–K transforms also demonstrates the compliance of the system with the linear systems theory (LST) [34].

After these analyses that demonstrate the viability of the EIS data, further analysis and model optimization using the genetic-inspired differential evolution (GDE) curve fitting (optimization) algorithm was performed [36,37].

## 4. Discussions

### 4.1. The Mixed Potential Model and Optimization of EIS Data

In this paper, for modeling the barrier layer formation on AA2098–T851 alloy, the mixed potential model (MPM) that was previously proposed by Macdonald et al. [28] was used. As explained previously, for investigating the electrochemical behavior of a highly electrochemically-active metal like Al, it is necessary to include both the partial cathodic process and the partial anodic process in the model, especially if the potential is made sufficiently negative that the cathodic partial reaction (e.g., hydrogen evolution) occurs to a significant extent. The MPM developed here combines the PDM that describes the passive state of the Al alloy with the Butler–Volmer equation that describes the partial cathodic reaction [28]. Optimization of the EIS data with MPM extracts PDM parameters and reveals kinetic information about the barrier layer formation and dissolution, as well as yielding values for the kinetic parameters for the cathodic partial process.

**Figure 7:**
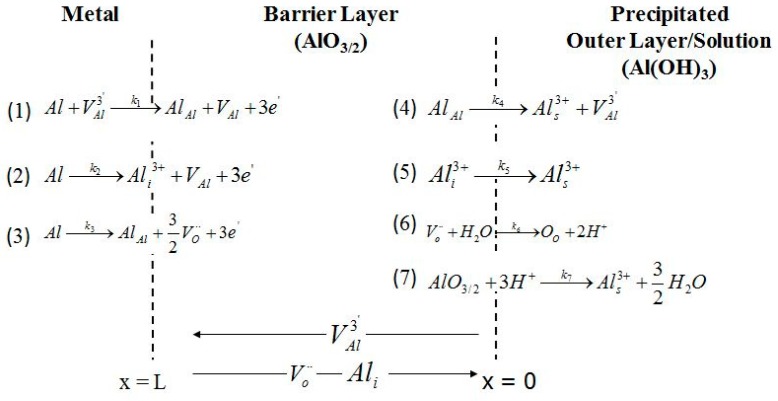
Point defect model representation. Al = metal atom, $V_{Al}^{3\prime }$ = cation vacancy on the metal sublattice, $Al_{Al}$ = metal cation in cation site, $V_{Al}$ = vacancy in metal phase, $Al_{i}^{3+}$ = interstitial cation, $\ddot{V_{O}}$ = oxygen vacancy on the oxygen sublattice, $Al_{s}^{3+}$ = metal cation in solution, O_O_ = oxygen anion on the oxygen sublattice of the barrier layer [33,38,39,40].

According to the PDM, the passive film comprises a bilayer structure of a highly-defective barrier layer that grows into the metal and a precipitated, porous layer that grows into the solution. Formation and dissolution of the protective barrier layer are described in terms of reactions with the rate constants (*k_i_*) that occur at the m/bl and the bl/ol interfaces, as shown in Figure 7 [33,38,39,40]. The value of the *k_i_* for the *i*th reaction in Figure 7 is defined by the constant parameters including the standard rate constants (*k_i_^0^*), transfer coefficients (*α_i_*), *a_i_*, *b_i_* and *c_i_* (defined in Table 1) as shown in Equation (2) [38].

(2) $$k_{i}=k_{i}^{0}e^{a_{i\;}E}{\;e}^{-b_{i}\;L}e^{c_{i}\;pH}\;$$

In this equation, *L* is the barrier layer film thickness and *E* is the applied potential.

Moreover, the semiconductor characteristics of the barrier layer that determine its electronic type (n-type or p-type) reveal more information about the reactions occurring during formation/dissolution of the barrier layer of the passive film [41]. The n-type character of the barrier layer of the passive film of Al [17,23,24,25,26,27] shows that the majority of the defects in the barrier layer on AA2098–T851 alloy are electron donors that are possibly oxygen vacancies and/or cation interstitials. We have not detected any p-type character, which indicates that cation vacancies, although undoubtedly present, are a minority defect. Therefore, Reactions (1) and (4) in Table 1, involving electron acceptors, were excluded in the optimization of the EIS results. According to PDM, the steady-state barrier layer thickness, *L_ss_*, regardless of the electronic character, is [38]:

(3) $$L_{ss}=\left(\frac{a_{7}-a_{3}}{b_{3}}\right)E+\left(\frac{c_{7}-c_{3}}{b_{3}}\right)pH+\frac{1}{b_{3}}ln\left[\frac{k_{7}^{0}}{k_{3}^{0}}{\left(\frac{C_{H}}{C_{H}^{0}}\right)}^{n}\right]$$

where $C_{H}$ is the hydrogen ion concentration at the f/s interface (M), $C_{H}^{0}$ is the concentration of hydrogen ion at standard state (1 M), *n* is the kinetic order of barrier layer dissolution with respect to hydrogen ion, and *a_i_*, *b_i_*, and *c_i_* are constants that are defined in Table 1 in terms of fundamental parameters [38]. Further, the PDM postulates that the steady- state partial anodic current density (*i_ss_*) can be calculated by [38]:

(4) $$i_{ss}=\delta F\left[k_{2}^{0}\;e^{a_{2\;}E}\;e^{b_{2}L_{ss}}\;e^{c_{2\;}pH}+k_{7\;}^{0}e^{a_{7\;}E}\;e^{c_{7\;}pH}{\left(\frac{C_{H}}{C_{H}^{0}}\right)}^{n}\right]$$

where *F* is Faraday’s constant (96,487 C mol^−1^) and *δ* is the stoichiometry of the metal in the solution. If no change in the oxidation state of the cation occurs upon ejection from the barrier layer into the solution at the bl/ol interface, *a_7_* = 0 and *a_3_E + b_3_L_ss_* = constant so that *i_ss_* would be potential independent.

As noted above, in the investigation of the barrier layer of the passive film by using the MPM, the partial cathodic current density (*i_cath_*) must be also considered in describing the passive state as the observed passive current density is *i_total_ = i_ss_ + i_cath_*. Applying the Butler–Volmer equation for an irreversible hydrogen evolution reaction (water reduction in deaerated condition is the dominant reduction reaction at pH > 4) yields the following expression for cathodic current density [42]:

(5) $$i_{cath}=-2Fk_{c}exp\left[-\frac{\alpha_{c}F}{RT}\left(E-E_{eq}\right)\right]$$

where *k_c_* is the rate constant of the cathodic reaction, *R* is the gas constant (8.314 J mol^−1^), *α_c_* is the transfer coefficient, *E* is the applied potential, and *E_eq_* is the equilibrium potential of the hydrogen electrode reaction in the solution. It is assumed that the fugacity of hydrogen ($f_{H_{2}}$) to be 10^−6^ in which case *E_eq_* is estimated to be approximately −240 mV_SHE_ at pH = 6.7. On this basis, we expect that the partial cathodic current density will be small but, nevertheless, significant.

**Figure 8:**
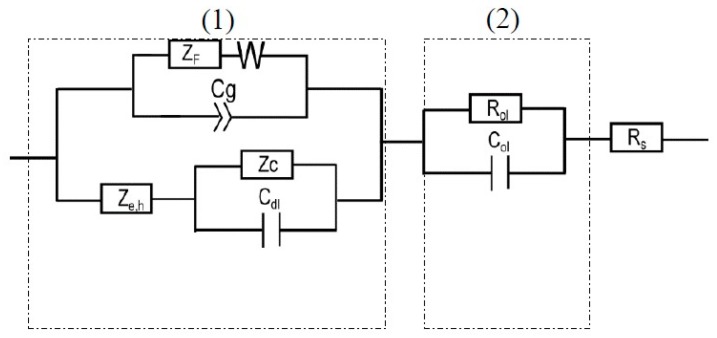
MPM equivalent circuit used for fitting EIS data of the passive layer on AA2098–T851 alloy in NaHCO_3_ solution in contact with 1 atm CO_2_.

The impedance expressions for the anodic and cathodic partial reactions were obtained by using Equations (4) and (5) (elaborated in Ref. [28,42]) which were then inserted into the electrical equivalent circuit (Figure 8) and used for optimization of the EIS results [21].

### 4.2. Quantum Tunneling Definition of Current Density

It is important to recognize that the cathodic reaction (hydrogen evolution) occurs upon the outer surface of the barrier layer; that is, at the bl/s (or bl/ol) interface. For that to occur, charge carriers must be transported across the barrier layer. Because the barrier layer is very thin (several nm), it is postulated that the transfer of charge across the barrier layer occurs by direct (resonant) or indirect quantum mechanical tunneling. Thus, numerous experimental studies have reported a decrease in the rates of redox reactions on passive metals and alloys coincident with increasing oxide film thickness and potential [16,43,44,45,46,47]. Further studies of the relationship between current density and potential reveal that these observations may be accounted for by quantum-mechanical tunneling theory developed originally by Gurney [29] and later by Gerischer [30]. That is, at a given potential *V*, the current density is defined by Equation (6) [29,30]:

(6) i∝∬n(E,V)N(E,x)P(E,x)dEdx

where n(E,V) is the density of electronic states having energy *E* in the metal, the density of acceptor states, N(E,x), also having energy *E* at a distance *x* from the metal surface in the solution phase (note that tunneling is an iso-energetic process), and the probability P(E,x) of electron tunneling through the barrier oxide thickness *L* from the reduced species in the solution to an acceptor state in the metal having identical energy. From quantum-mechanical theory, the tunneling probability is then written as [30]:

(7) $$P\left(E,x\right)\propto e^{-\frac{4\pi L}{h}\sqrt{2m_{e}\Delta E}}$$

where *h* is Planck’s constant and *m_e_* is the effective mass of the electron. The barrier layer thickness *L* can be calculated theoretically using the PDM and ΔE is the difference in Fermi level of electron in the solution and in the conduction band edge of the barrier layer oxide. Thus, the current density may be expressed as [48]:

(8) $$i=\hat{i_{0}}e^{-\hat{\beta }L}$$

where $\hat{i_{0}}$ is the film-free current density (i.e., when *L* = 0). From Equations (7) and (8), it is apparent that the tunneling constant can be expressed as:

(9) $$\hat{\beta }=\frac{4\pi }{h}\sqrt{2m_{e}\Delta E}.$$

According to the point defect model, [38,49,50,51] the thickness of the barrier oxide layer through which tunneling must occur can be written as:

(10) $$L_{ss}=\frac{1-\alpha }{\varepsilon }V+g$$

where ε is the electric field strength, and *g* is a function of pH and the standard rate constants for film formation at the metal/barrier layer (m/bl) interface and the dissolution rate at the bl/s interface, among other parameters (see Equation (3)). Substitution of Equation (10) into Equation (8) yields the tunneling current as:

(11) $$i=\hat{i_{0}}e^{-\hat{\beta }\left(\frac{1-\alpha }{\varepsilon }\right)V}e^{-\beta g}.$$

Equation (11) shows that the current is a function of voltage through the various parameters in the PDM and the quantum-mechanical tunneling probability. By substituting the equilibrium potential for the redox reaction (*E^e^*) into Equation (11), we can define the exchange current density as:

(12) $$i^{0}=\hat{i_{0}}e^{-\beta \left(\frac{1-\alpha }{\varepsilon }\right)E^{e}}e^{-\beta g}.$$

We emphasize that $\hat{i_{0}}$ is the exchange current density on the (hypothetical) bare metal surface; that is, the current density at the equilibrium potential in the absence of the barrier layer. Thus, Equation (12) allows calculation of the exchange current density of a redox couple on a passive metal from the exchange current density on a hypothetical film-free surface, using the tunneling probability, and the parameters of the PDM and vice versa. This is important, because the kinetic parameters (particularly the HER Tafel constant), which are often used to identify a particular reaction mechanism, are generally derived for a bare metal surface, such as that on Pt or Au. The application of this principle is shown below.

### 4.3. Equivalent Circuit for EIS Data Fitting

The equivalent circuit for interpretation of the EIS data for the AA2098–T851 alloy in NaHCO_3_ solution in a CO_2_ atmosphere based on the MPM is shown in Figure 8.

According to the PDM, the passive layer has a bilayer structure (the barrier layer and the precipitated outer layer) that are shown in Figure 8 by parts (1) and (2), respectively. The outer layer segment comprises the solution resistance between the RE and WE (*R_s_*) in series with the parallel resistance of the outer layer (*R_ol_*) with the capacitance of the outer layer (*C_ol_*). In this circuit, for the barrier layer (1), both anodic (based on the PDM) and cathodic (based on Butler–Volmer) reactions are considered. The anodic reaction is associated with *Z_F_* (the faradaic impedance), *C_g_* (the geometric constant phase element), and W (the Warburg impedance), which describes the movement of oxygen vacancy and/or aluminum interstitial point defects in the barrier layer. According to the PDM, due to the reactions in Figure 7, the value of *Z_F_* arises from the generation and/or annihilation of interfacial point defects, the rates of which are specified by the respective rate constants. Moreover, the accumulation of electronic charge between the metal and the bl/ol interface, due to the semiconductor characteristic of the non-homogeneous barrier layer, acts as a non-ideal capacitor and defines *C_g_*. Moreover, the Warburg impedance controls the transport of the electron donor point defects (oxygen vacancies or metal interstitials) across the barrier layer, as noted above. Additionally, in this circuit, the impedance of the partial cathodic reaction is represented by a Randles circuit comprising *R_c_*, (the charge transfer resistance of hydrogen evolution) and *C_dl_* (the double layer capacitance), previous work having shown that the impedance could be described by a non-reactive resistance. Finally, the last element in this circuit is *Z_e,h_* which is the impedance due to the movement of electronic defects across the barrier layer that by-passes *Z_F_* [42]. The detailed derivation of the impedance of each circuit elements in Figure 8, can be found in the literature [28,42].

### 4.4. Optimization Parameters

Using the Igor Pro software and by considering the MPM and the proposed equivalent circuit depicted in Figure 8, the interphasial model was optimized on the EIS data and values for all model parameters were extracted. The known, constant parameters used for the optimization are shown in Table 2.

**Figure 9:**
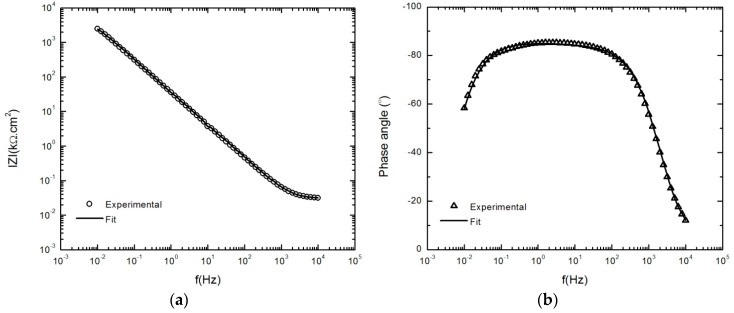
EIS measurements and model fits of the AA2098–T851 alloy after 24 h immersion at 300mVSCE in NaHCO_3_ solution in CO_2_ atmosphere at 25 °C. (**a**) Bode magnitude and (**b**) Bode phase plots.

After optimization, and using the parameter values so obtained, the calculated impedance spectra were compared with experimental data, as shown in Figure 9a,b. It can be seen that the calculated spectra match the experimental data very well.

**Figure 10:**
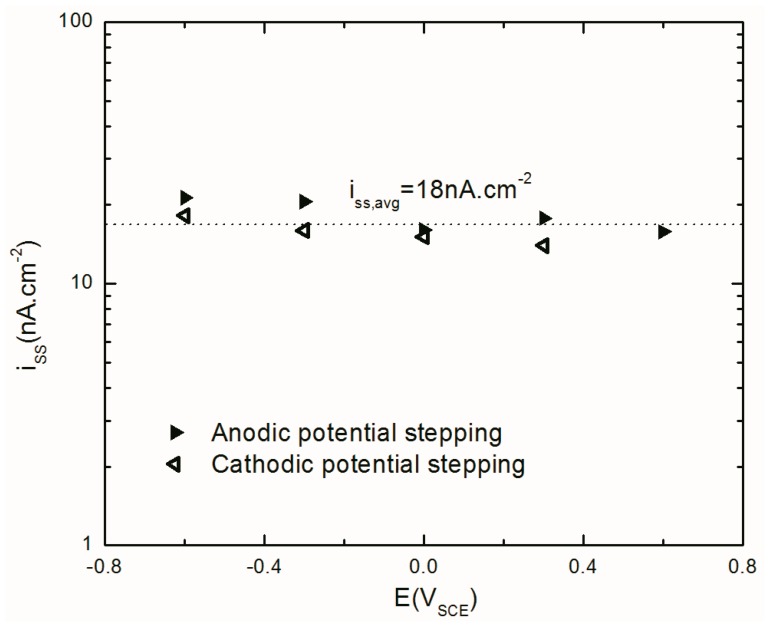
The steady-state anodic current density of AA2098–T851 alloy in 0.1M NaHCO_3_ after 24 h of polarization at different potentials in contact with 1 atm CO_2_ (pH = 6.7, 25 °C) during the anodic and cathodic potential stepping obtained from optimization of EIS results.

**Figure 11:**
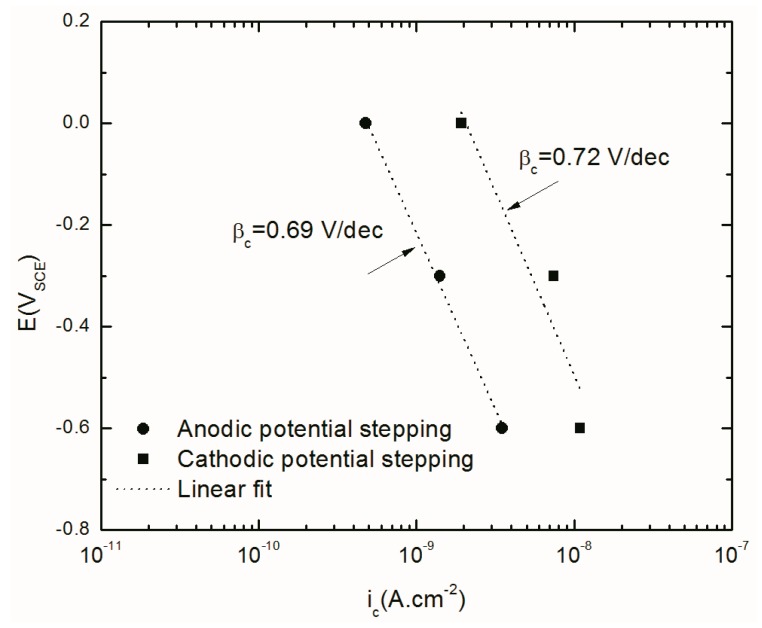
Current density of AA2098–T851 alloy in 0.1 M NaHCO_3_ after 24 h of polarization at different potentials in contact with 1 atm CO_2_ (pH = 6.7, 25 °C) during the anodic and cathodic potential stepping obtained from optimization of EIS results.

**Figure 12:**
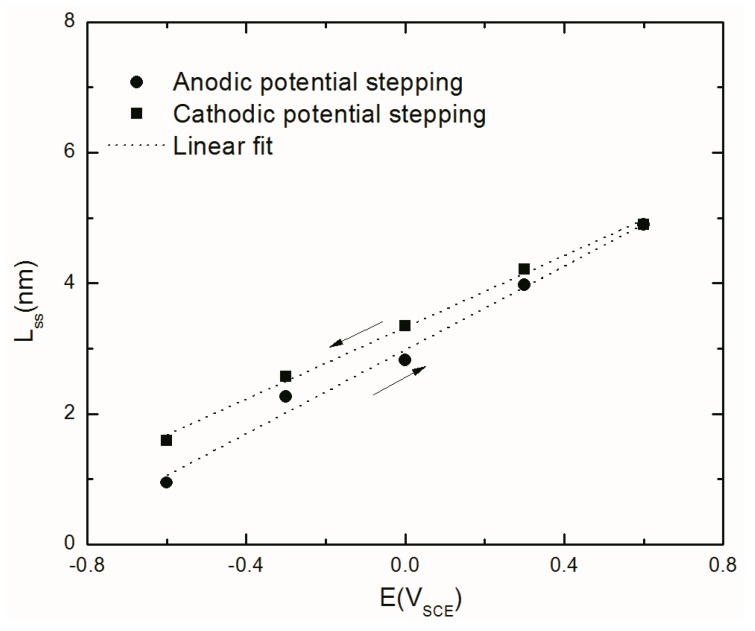
The steady-state thickness of the passive layer formed on AA2098–T851 alloy in 0.1M NaHCO_3_ after 24 h of polarization at different potentials in CO_2_ atmosphere (pH = 6.7, 25 °C) during the anodic and cathodic potential stepping obtained from optimization of EIS results [31].

**Figure 13:**
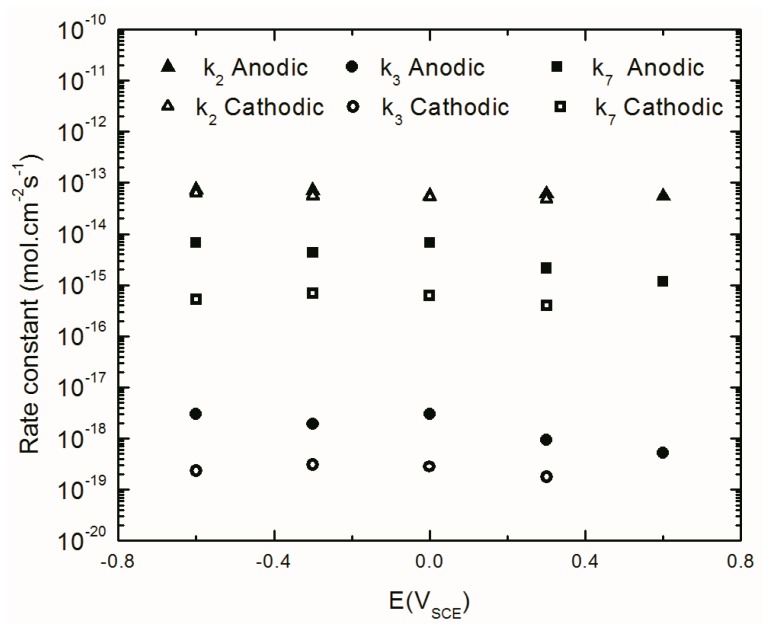
Rate constants for point defect reactions in Figure 7 obtained by MPM optimization of the EIS results at different stepping direction.

**Figure 14:**
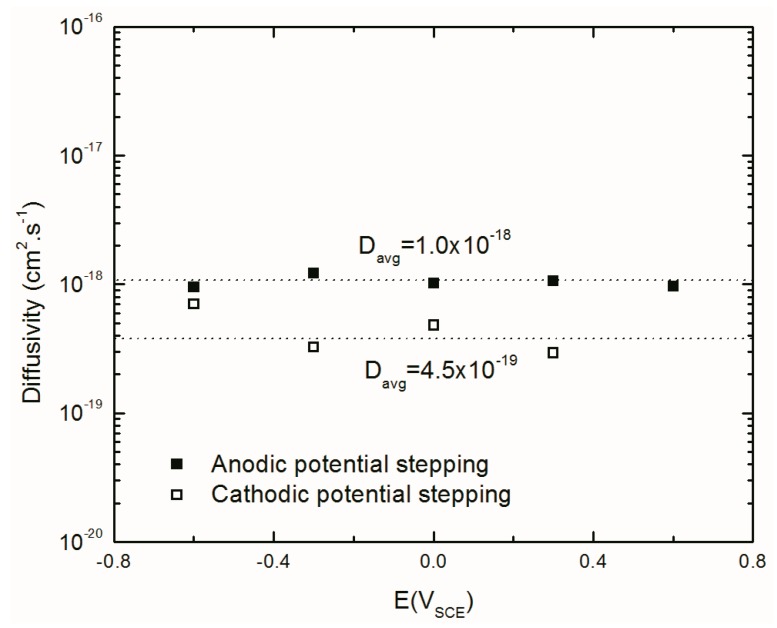
Calculated diffusivity of Al ion interstitials in the barrier layer with optimization of the EIS results at different potentials during anodic and cathodic potential stepping.

Also, the MPM variables and the kinetic parameters of the passive layer formation and dissolution reactions shown in Figure 7 were extracted (Table 3) and summarized in Figure 10, Figure 11, Figure 12, Figure 13 and Figure 14 (Only the optimization results of immediate interest are given in Table 3. For the whole set of fitting parameters please refer to the Table S1 in the supplementary materials). Examination of the data presented in Table 3 shows that at all potentials, *k*_2_ > *k*_3_, from which it is argued that the metal interstitial ($Al_{i}^{3+}$), rather than the oxygen vacancy ($V_{O}^{¨}$) is the dominant point defect in the barrier layer. Moreover, in Table 3 and displayed in Figure 12, the barrier layer thickness varies linearly with voltage, in conformity with Equation (3), and the postulated hysteresis in the thickness in the two potential stepping directions is quite apparent. The polarizability of the bl/s interface (*α*) is found to be constant (independent of voltage) as are the transfer coefficients and the standard rate constants show no systematic dependence on *E**_app_***. The independence of these fundamental parameters of the applied voltage is a requirement of electrochemical kinetic theory. Finally, the cation interstitial diffusivity also displays no systematic dependence on *E**_app_***, which is expected, because the diffusivity is a materials property.

#### 4.4.1. Steady-State Anodic Current Density

Figure 10 shows the anodic current density of AA2098–T851 alloy passive dissolution obtained from MPM optimization. Fitting results in this figure shows that the *i_ss_* with the mean value of 18 nA cm^−2^ is independent of the applied potential. This is in agreement with the PDM prediction for an n-type passive layer with δ=χ that is shown in Equation (1) [22].

Importantly, however, the fact that the small potential dependence of the passive current observed in Figure 2 is no longer apparent demonstrates that the dependence is due to the occurrence of the cathodic partial reaction at the bl/s interface. Moreover, no significant difference exists in the anodic partial current density for the two potential stepping directions. Considering Faraday’s law, the mean value of anodic current density was converted to the corrosion rate (C.R.) of 0.22 µm/yr.

#### 4.4.2. Cathodic Current Density

Figure 11 exhibits the Tafel relationship (straight line in semi-log plot) between partial cathodic current density and applied potential at both potential stepping directions. The values of the partial cathodic current density compared with the values of the partial anodic current density are small and therefore has only a minor effect in the total (observed) current values. However, the slope and linear dependence of the observed current density on the applied potential conforms with Tafel’s law, as shown in Figure 11. Moreover, the order of the hydrogen exchange current density on the oxide film (10^−8^ A.cm^−2^) that was calculated by using Equation (5) is in agreement with the reported value in the literature [52].

The linear fit in this graph indicates almost the same cathodic Tafel slope (hydrogen evolution reaction (HER) kinetic) for the anodic and cathodic potential stepping directions, with values of 0.72 V/dec and 0.69 V/dec, respectively, being determined. However, due to the potential-dependence of the barrier layer thickness and the quantum mechanical tunneling of charge carriers through the barrier layer, the kinetics of the HER on the passive surface are different from those on the film-free surface. Therefore, the observed Tafel constant may be corrected to the hypothetical film-free surface using the following equation [44,46,47,48,53,54,55,56,57,58]:

(13) $$\frac{2.303}{\hat{\beta_{c}}}=\frac{2.303}{\beta_{c}}+\frac{\hat{\beta }\left(1-\alpha \right)}{\varepsilon }$$

where $\hat{\beta_{c}}$ is the cathodic Tafel slope on film-free surface, $\hat{\beta }$ is the tunneling constant (0.58 × 10^8^ cm^−1^) [48] and *ε* is the electric field strength in b/l. Using the mean value of *β_c_* in both anodic and cathodic potential stepping directions as 0.7, $\hat{\beta_{c}}$ was calculated as 0.12V/dec. This is acceptably close to the value for the hydrogen evolution reaction on a bare metal surface (0.116) [59,60]. Table 4 shows some of the best known possible HER paths with their theoretical Tafel constant values.

The hydrogen evolution reaction on a bare metal is envisioned to proceed via two mechanisms with the first step being in common [59]:

(14) $$H_{2}O+e^{-}\to H_{ads}+OH^{-}$$

In the second step, the Tafel mechanism envisages hydrogen atom recombination:

(15) $$H+H\to H_{2}$$

whereas for the Abegg and Bodlander (Heyrovsky) mechanism envisages the ion-atom reaction [61]:

(16) $$H+H_{2}O+e^{-}\to H_{2}+OH^{-}$$

The two mechanisms being summarized to give the overall reaction as:

(17) $$2H_{2}O+2e^{-}\to H_{2}+2OH^{-}$$

The value of $\hat{\beta_{c}}$ obtained in this work is consistent with slow hydrogen atom discharge being the rate-determining step.

#### 4.4.3. Steady-State Thickness of the Barrier Layer

Figure 12 shows the steady-state thickness of the passive layer formed on AA2098–T851 alloy obtained from optimization of the EIS results using Equation (3).

This figure shows the linear dependence of the film thickness on the applied potential, which is in agreement with the prediction of the PDM. Additionally, different $\frac{\partial L_{ss}}{\partial E}$ values, (3.2 nm/V and 2.7 nm/V for anodic and cathodic potential stepping directions, respectively) reveal the irreversibility of the passivity that led into the slower rate of film thinning in the cathodic potential stepping direction than in the film growth rate when potential stepping in the anodic direction. This conclusion is in agreement with the calculated kinetic parameters obtained by the MPM as shown in Figure 13.

#### 4.4.4. The Kinetics of the Point Defect Reactions

The values obtained from the optimization, Table 3, show that the rate constants (*k_i_*) for reactions at the m/bl interface (*i* = 2, 3, 7) are independent of potential (because of compensation of the effects of voltage and bl thickness for *i* = 2 and 3, see below, and because for Reaction (7), δ = χ), the standard rate constants ($k_{i}^{0}$) and the transfer coefficients (*α_i_*), (*i* = 2, 3, 7) are independent of potential, (as required by fundamental electrochemical theory) and are also independent of potential stepping direction. Similar behavior has been observed and reported by others for carbon steel [21,28,42]. The potential independence of *k_i_* is accounted for as follows. According to the PDM, the rate constants for the generation and annihilation of the point defects at the interfaces in the steady-state can be written in general form as Equation (2), $k_{i}=k_{i}^{0}e^{a_{i}E}e^{b_{i}L_{ss}}e^{c_{i}pH},$ where $a_{i}$, $b_{i}$, and $c_{i}$ are defined in Table 1 in terms of fundamental quantities. From these definitions, together with Equation (3), we find that *a_i_E* + *b_i_L_ss_* = $2\left(1-\alpha \right)\gamma \chi -\alpha_{i}\beta \chi \gamma pH+\frac{\alpha_{i}}{\alpha_{3}}ln\left[\frac{k_{7}^{0}}{k_{3}^{0}}{\left(\frac{C_{H}}{C_{H}^{0}}\right)}^{n}\right]$, which is independent of potential for reactions occurring at the m/bl interface, thereby rendering the rate constants for Reactions (1)–(3) of Figure 7 also potential independent, as is seen in Figure 13. As was mentioned before, the rate constant for Reaction (7) of Figure 7, is potential independent by virtue of δ=χ.

Figure 13 shows the relationship between the rate constants (*k_i_*) for Reactions (3) and (7), as proposed in the PDM. These values were calculated in both anodic and cathodic potential stepping directions. Figure 13 shows that *k_7_* has higher values than *k*_3_, which is a demonstration of higher dissolution rate constant than that of the growth of the passive layer, but, of course, at steady-state the rates are equal. Moreover, this figure shows lower *k*_3_ and *k_7_* values during cathodic potential stepping direction compared to those in the opposite direction. This relationship between the kinetics of the point defect reactions has been also reported for the barrier layer of the passive film on carbon steel [21,28]. Because the thickness of the barrier layer is determined by Reactions (3) and (7) of Figure 7, regardless of the electronic character (i.e., regardless of what is the dominant defect and hence regardless of the rate constants for the other reactions), the irreversibility lies in the barrier layer thickness, as is evident from Figure 12.

Furthermore, $k_{2}$ (the rate constant for cation interstitial generation) is about four orders of magnitude higher than $k_{3}$ (the rate constant for oxygen generation vacancy), which has also been reported (by three orders of magnitude) for carbon steel [21]. Higher values of $k_{2}$ compared to the values of $k_{3}$ shows that the dominant point defects in the (bl) are cation ($Al_{i}^{3+}$) interstitials. This could be due to the larger radius of oxygen vacancies that require higher energy to be formed. Finally, it is also found that $k_{2}^{0}\approx k_{3}^{0}$ indicating that the differences in the rate constants for Reactions (2) and (3) lie in the properties of the barrier layer and not in the fundamental processes that occur along the reaction coordinate.

#### 4.4.5. Diffusivity of Aluminum Interstitials in the Barrier Layer

As explained above, the Al interstitials are the dominant point defect in the barrier layer of the passive film. Based on the PDM, due to the electric field (ε) in the barrier layer, Al interstitials migrate from the m/bl interface toward the bl/s interface. The diffusivity of these defects (*D*) at different potentials can be calculated from the expression [62]:

(18) $$\sigma =\frac{1}{I_{ss}}\sqrt{\frac{D}{2}}\times \frac{\varepsilon }{1-\alpha }$$

where σ is the Warburg coefficient, *I_ss_* is the steady-state anodic current density (A/cm^2^) and *ε* is the electric field strength within the barrier layer (3 × 10^6^ Vcm^−1^).

Figure 14 shows the values of *D* as a function of applied potential. This figure indicates that, as expected, *D* is potential independent and the mean value of *D* for anodic and cathodic potential stepping directions are 1.0 × 10^−18^ and 4.5 × 10^−19^ cm^2^/s respectively. Higher values of *D* in the anodic potential stepping direction compared to that in cathodic potential stepping direction (~2 times), could be due to the retention of some aspect of the defect structure induced on anodic potential stepping that is not reversed upon cathodic potential stepping [28]. This observation has also been reported for carbon steel [21,28].

## 5. Conclusions

In this work, the kinetics of the passive layer formation on AA2098–T851 alloy in 0.1 M NaHCO_3_ in a CO_2_ atmosphere of 1 atm pressure at 25 °C was investigated. The EIS results confirm the formation of a passive layer having lower capacitance when formed by potentiostatic polarization of the alloy in the anodic potential stepping direction than when observed by subsequent stepping of the potential in the cathodic direction. This dependence of the kinetics of the passivity on the potential stepping direction is attributed to irreversible changes in the point defect structure of the barrier layer. Additionally, through optimization of the MPM on the experimental EIS results, the contribution of the cathodic partial current to the total current was determined to be small. Optimization of the MPM on the EIS data confirmed the irreversibility of the passivation of the AA2098–T851 alloy. Additionally, extracted MPM parameters showed that the anodic current is potential-independent, as predicted by the point defect model for an n-type barrier layer of the passive film. The calculated value of the passive current density leads to a more accurate calculation of the corrosion rate. Moreover, it has been shown that the Al interstitial comprise the majority of the point defects in the barrier layer of AA2098–T851 alloy. Finally, by using a mixed potential model that incorporates quantum mechanical tunneling of charge carriers through the barrier oxide layer on the passive metal surface we have been able to recover a cathodic Tafel constant that is in accord with that predicted for the HER reaction (the slow proton discharge mechanism).

## Figures and Tables

**Table 1:** Coefficients for the rate constants for the reactions that generate and annihilate point defects at the m/bl interface (Reactions (1)–(3)) and at the bl/s interface (Reactions (4)–(6)) in Figure 7 and for dissolution of the film (Reaction (7)) [33,38,39,40]. $K_{i}=K_{i}^{0}e^{a_{i}V}e^{b_{i}L}e^{c_{i}pH}$, *K = εγ, γ = F/RT*.

Reaction	*a_i_* (V^−1^)	*b_i_* (cm^−1^)	*c_i_*	Units of $K_{i}^{0}$
(1) $m+V_{M}^{\chi^{\prime }}\overset{k_{1}}{\to }M_{M}+v_{m}+\chi e^{\prime }$	*α*_1_(1 − *α*)*χγ*	−*α*_1_*βχK*	−*α*_1_*βχγ*	$\frac{\mathrm{cm}}{s}$
(2) $m\overset{k_{2}}{\to }M_{i}^{\chi +}+v_{m}+\chi e\prime$	*α*_2_(1 − *α*)*χγ*	−*α*_2_*βχK*	−*α*_2_*βχγ*	$\frac{\mathrm{mol}}{{\mathrm{cm}}^{2}s}$
(3) $m\overset{k_{3}}{\to }M_{M}+\chi /2\ddot{V_{O}}+\chi e\prime$	*α*_3_(1 − *α*)*χγ*	−*α*_3_*βχK*	−*α*_3_*βχγ*	$\frac{\mathrm{mol}}{{\mathrm{cm}}^{2}s}$
(4) $M_{M}\overset{k_{4}}{\to }M^{\delta +}+\left(\delta -\chi \right)e\prime$	*α*_4_ *αδγ*	-	*α*_4_ *βδγ*	$\frac{\mathrm{mol}}{{\mathrm{cm}}^{2}s}$
(5) $M_{i}^{\chi +}\overset{k_{5}}{\to }M^{\delta +}+\left(\delta -\chi \right)e\prime$	*α*_5_ *αδγ*	-	*α*_5_ *βδγ*	$\frac{\mathrm{cm}}{s}$
(6) $\ddot{V_{O}}+H_{2}O\overset{k_{6}}{\to }O_{O}+2H^{+}$	2*α*_6_*αγ*	-	2*α*_6_*βδγ*	$\frac{\mathrm{cm}}{s}$
(7) $MO_{\chi /2}+\chi H^{+}\overset{k_{7}}{\to }M^{\delta +}+\chi /2\;H_{2}O+\left(\delta -\chi \right)e\prime$	*α*_7_*α*(*δ − χ*) *γ*	-	*α*_7_(*δ − χ*)*βγ*	$\frac{\mathrm{mol}}{{\mathrm{cm}}^{2}s}$

**Table 2:** Known, constant parameter used in optimization of EIS data with their sources.

Parameter	Value	Unit	Source
Dependence of the potential drop across bl/ol upon pH (*β*)	−0.014	V	Calculated from experiment
Electric Field Strength (*ε*)	3 × 10^6^	V cm^−1^	Assumed
Oxidation state (*χ*)	3		Assigned
Molar volume of oxide per cation(*Ω*)	12.91	cm^3^ mol^−1^	From density
Kinetic order of Reaction (7) (*n*)	0.5	-	Assumed
Oxidation state (*δ*)	+3	-	Assigned
Solution resistance (*Rs*)	30	Ω	From EIS

**Table 3:** Kinetic parameters of passive layer formation obtained by optimization of the EIS results based on the MPM [31].

Anodic Potential Stepping Direction						Cathodic Potential Stepping Direction			
*E_app_ (V_SCE_)*	−0.6	−0.3	0	0.3	0.6	0.3	0	−0.3	−0.6
*α*	0.18	0.18	0.18	0.18	0.18	0.18	0.18	0.18	0.18
*α*_2_	0.15	0.11	0.12	0.11	0.11	0.11	0.11	0.11	0.11
*α*_3_	0.29	0.25	0.27	0.25	0.23	0.25	0.25	0.24	0.26
*α_c_*	0.18	0.179	0.179	-	-	-	0.16	0.18	0.18
*k*^0^_2_ (mol cm^−2^.s)	5.3 × 10^−10^	4.3 × 10^−10^	2.8 × 10^−10^	3.0 × 10^−10^	5.8 × 10^−10^	8.9 × 10^−10^	6.3 × 10^−10^	7.9 × 10^−10^	8.8 × 10^−10^
*k*^0^_3_ (mol.cm^−2^.s)	1.1 × 10^−10^	8.5 × 10^−10^	4.9 × 10^−10^	5.8 × 10^−10^	1.0 × 10^−10^	7.6 × 10^−10^	8.2 × 10^−10^	9.3 × 10^−10^	5.2 × 10^−10^
*k*^0^_7_ (mol cm^−2^.s)	6.8 × 10^−15^	4.3 × 10^−15^	6.8 × 10^−15^	2.1 × 10^−15^	1.2 × 10^−15^	4.0 × 10^−16^	6.3 × 10^−16^	6.9 × 10^−16^	5.3 × 10^−16^
*k*_2_ (mol cm^−2^.s)	7.4 × 10^−14^	7.1 × 10^−14^	5.6 × 10^−14^	6.2 × 10^−14^	5.5 × 10^−14^	4.8 × 10^−14^	5.2 × 10^−14^	5.5 × 10^−14^	6.3 × 10^−14^
*k*_3_ (mol cm^−2^.s)	3.0 × 10^−18^	1.9 × 10^−18^	3.0 × 10^−18^	9.5 × 10^−19^	5.3 × 10^−19^	1.8 × 10^−19^	2.8 × 10^−19^	3.1 × 10^−19^	2.4 × 10^−19^
*k_7_* (mol cm^−2^ s)	6.8 × 10^−15^	4.3 × 10^−15^	6.8 × 10^−15^	2.1 × 10^−15^	1.2 × 10^−15^	4.0 × 10^−16^	6.3 × 10^−16^	6.9 × 10^−16^	5.3 × 10^−16^
*k_C_* (mol cm^−2^.s)	2.8 × 10^−14^	9.4 × 10^−14^	2.7 × 10^−13^	-	-	-	7.2 × 10^−13^	5.2 × 10^−13^	8.7 × 10^−14^
*D* (cm^2^ s^−1^)	9.5 × 10^−19^	1.2 × 10^−18^	1.0 × 10^−18^	1.1 × 10^−18^	9.7 × 10^−19^	2.9 × 10^−19^	4.8 × 10^−19^	3.3 × 10^−19^	7.0 × 10^−19^
*I_ss_* (nA cm^−2^)	21.3	20.6	16.1	17.8	15.8	14.0	15.1	16.0	18.2
*I_c_* (nA cm^−2^)	−3.5	−1.4	−0.47	-	-	-	−1.9	−7.4	−10.9
*L_ss_* (nm)	0.95	2.26	2.82	3.97	4.90	4.21	3.35	2.57	1.59

**Table 4:** Rate-determining steps in hydrogen evolution reaction (HER) and corresponding Tafel slopes [57,58].

Rate-Determining Step	Mechanism	HER Tafel Slope (V/dec)
$\left(I\right)\;\;\;H_{2}O+e^{-}\to H_{ads}+OH^{-}$	The Slow Hydrogen Discharge	0.116
$\left(\mathrm{II}\right)\;\;H+H\to H_{2}$	Atomic Hydrogen	0.029
$\left(\mathrm{III}\right)\;H+H_{2}O+e^{-}\to H_{2}+OH^{-}$	Electrochemical Mechanism	0.038

## Supplementary Materials

The following are available online at https://www.mdpi.com/1996-1944/12/12/1912/s1, Table S1: Fitting parameters of passive layer formation obtained by optimization of the EIS results based on the MPM.

## References

[1] Starke E.A., Staley J.T. (1996). Application of modern aluminum alloys to aircraft. Prog. Aerosp. Sci..

[2] Kumar A., Prasad R.K., Dwarakadasa E.S. (1991). Anisotropic tensile and fracture behaviour of aluminum lithium alloy 8090. Met. Mater. Process..

[3] Criner C.B. (1957). Aluminum Base Alloy. U.S. Patent.

[4] Criner C.B. (1959). Aluminum Base Alloy. U.S. Patent.

[5] Palit G.C., Elayaperumal K. (1978). Passivity and pitting of corrosion resistant pure metals Ta, Nb, Ti, Zr, Cr and A1 in chloride solutions. Corros. Sci..

[6] Ilevbare G.O., Scully J.R., Yuan J., Kelly R.G. (2000). Inhibition of pitting corrosion on aluminum alloy 2024-T3: Effect of soluble chromate additions vs chromate conversion coating. Corrosion.

[7] Buchheit R.G., Grant R.P., Hlava P.F., McKenzie B., Zender G.L. (1997). Local dissolution phenomena associated with S phase (Al2CuMg) particles in aluminum alloy 2024-T3. J. Electrochem. Soc..

[8] Chen G.S., Gao M., Wei R.P. (1996). Microconstituent-induced pitting corrosion in aluminum alloy 2024-T3. Corrosion.

[9] Muller I.L., Galvele J.R. (1977). Pitting potential of high purity binary aluminium alloys—I. Al Cu alloys. Pitting and intergranular corrosion. Corros. Sci..

[10] Scully J.R., Peebles D.E., Romig A.D., Frear D.R., Hills C.R. (1992). Metallurgical factors influencing the corrosion of aluminum, Al-Cu, and Al-Si alloy thin films in dilute hydrofluoric solution. Metall. Trans. A.

[11] Mears R.B., Brown R.H. (1941). Causes of corrosion currents. Ind. Eng. Chem..

[12] Frankel G.S. (1998). Pitting corrosion of metals a review of the critical factors. J. Electrochem. Soc..

[13] Lei X., Saatchi A., Ghanbari E., Dang R., Li W., Wang N., Macdonald D.D. (2019). Studies on Pitting Corrosion of Al-Cu-Li Alloys Part I: Effect of Li Addition by Microstructural, Electrochemical, In-situ, and Pit Depth Analysis. Materials.

[14] Ghanbari E., Saatchi A., Lei X., Macdonald D.D. (2019). Studies on Pitting Corrosion of Al-Cu-Li Alloys Part II: Breakdown Potential and Pitting Initiation. Materials.

[15] Verwey E. (1935). Electrolytic conduction of a solid insulator at high fields The formation of the anodic oxide film on aluminium. Physica.

[16] Vetter K.J., Gorn F. (1973). Kinetics of layer formation and corrosion processes of passive iron in acid solutions. Electrochim. Acta.

[17] Zhang B., Li Y., Wang F. (2007). Electrochemical corrosion behaviour of microcrystalline aluminium in acidic solutions. Corros. Sci..

[18] Zhao X. (2006). Exfoliation Corrosion Kinetics of High Strength Aluminum Alloys. Ph.D. Thesis.

[19] Chao C.Y., Lin L.F., Macdonald D.D. (1981). A point defect model for anodic passive films I. Film growth kinetics. J. Electrochem. Soc..

[20] Lin L.F., Chao C.Y., Macdonald D.D. (1981). A point defect model for anodic passive films II. Chemical breakdown and pit initiation. J. Electrochem. Soc..

[21] Lu P., Sharifi-Asl S., Kursten B., Macdonald D.D. (2015). The Irreversibility of the Passive State of Carbon Steel in the Alkaline Concrete Pore Solution under Simulated Anoxic Conditions. J. Electrochem. Soc..

[22] Macdonald D.D., Biaggio S.R., Song H. (1992). Steady State Passive Films Interfacial Kinetic Effects and Diagnostic Criteria. J. Electrochem. Soc..

[23] Martin F.J., Cheek G.T., O’Grady W.E., Natishan M.P. (2005). Impedance studies of the passive film on aluminium. Corros. Sci..

[24] Fernandes J.C.S., Picciochi R., Belo M.D.C., Silva T.M.E., Ferreira M.G.S.E., Fonseca I.T. (2004). Capacitance and photoelectrochemical studies for the assessment of anodic oxide films on aluminium. Electrochim. Acta.

[25] McCafferty E. (2003). Semiconductor aspects of the passive oxide film on aluminum as modified by surface alloying. Corros. Sci..

[26] Bockris J.O., Kang Y. (1997). The protectivity of aluminum and its alloys with transition metals. J. Solid State Electrochem..

[27] Chang C.L., Sankaranarayanan S.K., Engelhard M.H., Shutthanandan V., Ramanathan S. (2009). On the Relationship between Nonstoichiometry and Passivity Breakdown in Ultrathin Oxides: Combined Depth-Dependent Spectroscopy, Mott− Schottky Analysis, and Molecular Dynamics Simulation Studies. J. Phys. Chem. C.

[28] Lu P., Kursten B., Macdonald D.D. (2014). Deconvolution of the Partial Anodic and Cathodic Processes during the Corrosion of Carbon Steel in Concrete Pore Solution under Simulated Anoxic Conditions. Electrochim. Acta.

[29] Gurney R.W., Fowler R.H. (1932). The quantum mechanics of electrochemistry. II. Proc. R. Soc. A. Math. Phys. Eng. Sci..

[30] Gerischer H. (1960). Kinetics of oxidation-reduction reactions on metals and semiconductors. I. General remarks on the electron transition between a solid body and a reduction-oxidation electrolyte. Z. Phys. Chem. Neue Folge.

[31] Ghanbari E., Saatchi A., Lei X., Kovalov D., Macdonald D.D. Passivity Breakdown and Pitting Corrosion of Al- Li Aerospace Alloys. Proceedings of the DoD Allied Nations Technical Corrosion Conference.

[32] JSun B., Zhang G.A., Liu W., Lu M.X. (2012). The formation mechanism of corrosion scale and electrochemical characteristic of low alloy steel in carbon dioxide-saturated solution. Corros. Sci..

[33] Macdonald D.D. (2006). Reflections on the history of electrochemical impedance spectroscopy. Electrochim. Acta.

[34] Macdonald D.D., Urquidi-Macdonald M. (1985). Application of Kramers-Kronig Transforms in the Analysis of Electrochemical Systems I. Polarization Resistance. J. Electrochem. Soc..

[35] Urquidi-Macdonald, Mirna S.R., Macdonald D.D. (1990). Applications of Kramers—Kronig transforms in the analysis of electrochemical impedance data—III. Stability and linearity. Electrochim. Acta.

[36] Ellis 2: Complex curve fitting for one independent variable IgorExchange. http://www.igorexchange.com/project/Ellis2.

[37] GenCurvefit | IgorExchange. http://www.igorexchange.com/project/gencurvefit.

[38] Macdonald D.D. (1992). The point defect model for the passive state. J. Electrochem. Soc..

[39] Macdonald D.D. (2006). On the existence of our metals-based civilization I. Phase-space analysis. J. Electrochem. Soc..

[40] Macdonald D.D. (1999). Passivity–the key to our metals-based civilization. Pure Appl. Chem..

[41] Macdonald D.D., Urquidi-Macdonald M. (1990). Theory of Steady-State Passive Films. J. Electrochem. Soc..

[42] Sharifi-Asl S., Taylor M.L., Lu Z., Engelhardt G.R., Kursten B., Macdonald D.D. (2013). Modeling of the electrochemical impedance spectroscopic behavior of passive iron using a genetic algorithm approach. Electrochim. Acta.

[43] Vetter K.J., Schultze J.W. (1973). Tunnelübergang der Elektronen und thermische Aktivierung bei der anodischen Sauerstoffentwicklung., Berichte Der Bunsengesellschaft Für Phys. Chemie.

[44] Moffat T.P., Yang H., Fan F.R.F., Bard A.J. (1992). Electron-Transfer Reactions on Passive Chromium. J. Electrochem. Soc..

[45] Hamann C.H. (1971). Die Oxidation von Kohlenmonoxid an Platin im sauren Elektrolyten., Berichte Der Bunsengesellschaft Für Phys. Chemie.

[46] Schultze J.W., Vetter K.J. (1973). The influence of the tunnel probability on the anodic oxygen evolution and other redox reactions at oxide covered platinum electrodes. Electrochim. Acta.

[47] Schuldiner S. (1968). Oxidation of hydrogen on a passive platinum electrode. J. Electrochem. Soc..

[48] Bao J., Macdonald D.D. (2007). Oxidation of hydrogen on oxidized platinum: Part I: The tunneling current. J. Electroanal. Chem..

[49] Macdonald D.D. (2012). Some personal adventures in passivity—A review of the point defect model for film growth. Elektrokhimiya.

[50] Macdonald D.D., Engelhardt G.R. (2010). The point defect model for bilayer passive films. ECS Trans..

[51] Macdonald D.D. (2011). The history of the point defect model for the passive state: A brief review of film growth aspects. Electrochim. Acta.

[52] Vijh A.K. (1969). Electrolytic hydrogen evolution reaction on aluminum, oxide-covered electrodes. J. Phys. Chem..

[53] Torresi R.M., Camara O.R., de Pauli C.P., Giordano M.C. (1987). Hydrogen evolution reaction on anodic titanium oxide films. Electrochim. Acta.

[54] Boodts J.C.F., Trasatti S. (1989). Hydrogen evolution on iridium oxide cathodes. J. Appl. Electrochem..

[55] Dickinson T., Greef R., Wynne-Jones L. (1969). The kinetics of the chlorine electrode reaction at a platinum electrode. Electrochim. Acta.

[56] Schmickler W. (1977). Resonance tunnelling through oxide layers on passive iron electrodes. J. Electroanal. Chem. Interfacial. Electrochem.

[57] Schmickler W., Ulstrup J. (1977). A theory of electron transfer reactions at film-covered metal electrodes. Chem. Phys..

[58] Gerischer H. (1990). The impact of semiconductors on the concepts of electrochemistry. Electrochim. Acta.

[59] Bockris J., Potter E.C. (1952). The mechanism of the cathodic hydrogen evolution reaction. J. Electrochem. Soc..

[60] Pentland N., Bockris J., Sheldon E. (1957). Hydrogen evolution reaction on copper, gold, molybdenum, palladium, rhodium, and iron mechanism and measurement technique under high purity conditions. J. Electrochem. Soc..

[61] Heyrovský J. (1927). A theory of overpotential. Recl. Des Trav. Chim. Des Pays-Bas..

[62] Chao C.Y., Lin L.F., Macdonald D.D. (1982). A point defect model for anodic passive films III. Impedance response. J. Electrochem. Soc..

