# Supplementary material for: Studies on Pitting Corrosion of Al–Cu–Li Alloys Part III: Passivation Kinetics of AA2098–T851 Based on the Point Defect Model
Source: Materials (Basel). 2019 Jun 13;12(12):1912. doi: 10.3390/ma12121912 (PMC6631545; doi:10.3390/ma12121912)
Supplement: Supplementary file 1 [file materials-12-01912-s001.pdf]

materials

Supplementary Materials: Studies on Pitting Corrosion of Al–Cu–Li Alloys Part III: Passivation Kinetics of AA2098–T851 Based on the Point Defect Model

Elmira Ghanbari, Alireza Saatchi, Xiaowei Lei and Digby D. Macdonald

Table S1. Fitting parameters of passive layer formation obtained by optimization of the EIS results based on the MPM.

	Anodic Potential Stepping Direction					Cathodic Potential Stepping Direction			
$E_{app}(V_{SCE})$	−0.6	−0.3	0	0.3	0.6	0.3	**0**	−0.3	−0.6
α	0.18	0.18	0.18	0.18	0.18	0.18	0.18	0.18	0.18
α_2	0.15	0.11	0.12	0.11	0.11	0.11	0.11	0.11	0.11
α_3	0.29	0.25	0.27	0.25	0.23	0.25	0.25	0.24	0.26
α_c	0.18	0.179	0.179	-	-	-	0.16	0.18	0.18
k^0_2 (mol.cm^{-2}.s)	5.3×10^{-10}	4.3×10^{-10}	2.8×10^{-10}	3.0×10^{-10}	5.8×10^{-10}	8.9×10^{-10}	6.3×10^{-10}	7.9×10^{-10}	8.8×10^{-10}
k^0_3 (mol.cm^{-2}.s)	1.1×10^{-10}	8.5×10^{-10}	4.9×10^{-10}	5.8×10^{-10}	1.0×10^{-10}	7.6×10^{-10}	8.2×10^{-10}	9.3×10^{-10}	5.2×10^{-10}
k^0_7 (mol.cm^{-2}.s)	6.8×10^{-15}	4.3×10^{-15}	6.8×10^{-15}	2.1×10^{-15}	1.2×10^{-15}	4.0×10^{-16}	6.3×10^{-16}	6.9×10^{-16}	5.3×10^{-16}
k_2 (mol.cm^{-2}.s)	7.4×10^{-14}	7.1×10^{-14}	5.6×10^{-14}	6.2×10^{-14}	5.5×10^{-14}	4.8×10^{-14}	5.2×10^{-14}	5.5×10^{-14}	6.3×10^{-14}
k_3 (mol.cm^{-2}.s)	3.0×10^{-18}	1.9×10^{-18}	3.0×10^{-18}	9.5×10^{-19}	5.3×10^{-19}	1.8×10^{-19}	2.8×10^{-19}	3.1×10^{-19}	2.4×10^{-19}
k_7 (mol.cm^{-2}.s)	6.8×10^{-15}	4.3×10^{-15}	6.8×10^{-15}	2.1×10^{-15}	1.2×10^{-15}	4.0×10^{-16}	6.3×10^{-16}	6.9×10^{-16}	5.3×10^{-16}
k_C (mol.cm^{-2}.s)	2.8×10^{-14}	9.4×10^{-14}	2.7×10^{-13}	-	-	-	7.2×10^{-13}	5.2×10^{-13}	8.7×10^{-14}
D (cm^2.s^{-1})	9.5×10^{-19}	1.2×10^{-18}	1.0×10^{-18}	1.1×10^{-18}	9.7×10^{-19}	2.9×10^{-19}	4.8×10^{-19}	3.3×10^{-19}	7.0×10^{-19}
I_{ss} (nA.cm^{-2})	21.3	20.6	16.1	17.8	15.8	14.0	15.1	16.0	18.2
I_c (nA.cm^{-2})	−3.5	−1.4	−0.47	-	-	-	-1.9	-7.4	-10.9
L_{ss} (nm)	0.95	2.26	2.82	3.97	4.90	4.21	3.35	2.57	1.59
CPE_g (F.cm^{-2})	5.7×10^{-6}	4.6×10^{-6}	3.8×10^{-6}	3.3×10^{-6}	$2. 9 \times 10^{-6}$	3.1×10^{-6}	3.5×10^{-6}	3.9×10^{-6}	4.8×10^{-6}
$CPE_g{}^P$	0.96	0.95	0.95	0.96	0.96	0.96	0.96	0.96	0.96
R_{eh} (Ω.cm^2)	2.4×10^8	3.2×10^8	1.0×10^8	3.8×10^8	1.9×10^9	4.4×10^8	2.3×10^8	1.4×10^8	2.2×10^8
C_{dl} (F.cm^{-2})	9.6×10^{-8}	6.4×10^{-8}	1.5×10^{-8}	2.2×10^{-8}	2.3×10^{-8}	1.2×10^{-8}	1.5×10^{-8}	2.6×10^{-8}	8.1×10^{-8}
R_{ol} (Ω.cm^2)	26000	58584	40830	29417	90900	85367	95493	35237	35112
C_{ol} (F.cm^{-2})	9×10^{-4}	6.8×10^{-4}	5.1×10^{-4}	4.9×10^{-4}	3.6×10^{-4}	3.2×10^{-4}	2.4×10^{-4}	4.9×10^{-4}	7.5×10^{-4}
R_s (Ω.cm^2)	30	30	30	30	30	30	30	30	30

© 2019 by the authors. Submitted for possible open access publication under the terms and conditions of the Creative Commons Attribution (CC BY) license (http://creativecommons.org/licenses/by/4.0/).
